# 394. Impact of Misdiagnosis of *Clostridioides difficile* Infection (CDI) by Standard-of-care Specimen Collection and Testing on Estimates of Hospitalized CDI Incidence Among Adults in Louisville, Kentucky, 2019-2020

**DOI:** 10.1093/ofid/ofac492.472

**Published:** 2022-12-15

**Authors:** Julio A Ramirez, Frederick Angulo, Ruth Carrico, Stephen Furmanek, Senen Pena Oliva, Joann M Zamparo, Elisa Gonzalez, Pingping Zhang, Leslie Wolf Parrish, Subathra Marimuthu, Michael W Pride, Sharon Gray, Catia Matos Ferreira, Forest W Arnold, Raul E Isturiz, Nadia Minarovic, Jennifer Moisi, Luis Jodar

**Affiliations:** Norton Healthcare, Louisville, Kentucky; Pfizer Vaccines, Collegeville, Pennsylvania; Norton Healthcare, Louisville, Kentucky; Norton Healthcare, Louisville, Kentucky; School of Medicine, University of Louisville, Louisville, Kentucky; Pfizer Vaccines, Collegeville, Pennsylvania; Pfizer Vaccines, Collegeville, Pennsylvania; Pfizer Vaccines, Collegeville, Pennsylvania; School of Medicine, University of Louisville, Louisville, Kentucky; School of Medicine, University of Louisville, Louisville, Kentucky; Pfizer Vaccines, Collegeville, Pennsylvania; Pfizer Vaccines, Collegeville, Pennsylvania; Pfizer Vaccines, Collegeville, Pennsylvania; University of Louisville School of Medicine, Louisville, Kentucky; Pfizer Vaccines, Collegeville, Pennsylvania; Pfizer Vaccines, Collegeville, Pennsylvania; Pfizer Vaccines, Collegeville, Pennsylvania; Pfizer Vaccines, Collegeville, Pennsylvania

## Abstract

**Background:**

Public health surveillance indicates that there is a high population-based incidence of laboratory-confirmed hospitalized CDI cases in the United States. Although reported CDI cases are identified via standard-of-care (SOC) specimen collection and CDI testing practices, the impact of SOC misdiagnosis on the reported CDI incidence is uncertain.

**Methods:**

Active surveillance from Oct 14, 2019, to Apr 11, 2020, identified inpatients aged ≥50 years with diarrhea (≥3 stools with Bristol score ≥5 in 24 hours) at all wards at 8 of the 9 adult hospitals in Louisville, Kentucky (population >50 years = 276 456). Study stool specimens from inpatients with diarrhea were screened by rapid GDH/toxin membrane enzyme immunoassay and the positive samples tested by PCR and cell cytotoxicity neutralization assay (CCNA). A study CDI case was a patient with PCR positive/CCNA positive stool or PCR positive stool with pseudomembranous colitis (PMC). Incidence (non-recurrent CDI cases/100 000 persons aged >50 years per year [PY]) was adjusted for the hospitalization share of participating hospitals and, in a sensitivity analysis, for patients with diarrhea without a CDI test result. SOC stool specimen CDI testing occurred independent of the study.

**Results:**

Among 1541 inpatients with diarrhea, study testing identified 109 non-recurrent CDI cases; 18 (16.5%) had PMC, 36 (33.0%) were admitted to intensive care, and 21 (19.3%) died during the 90-day follow-up. Study hospitalized CDI incidence was 154/100 000 PY (202/100 000 PY in the sensitivity analysis). SOC hospitalized CDI incidence was 121/100 000 PY. Of the 109 study CDI cases, 44 (40%) were not SOC-diagnosed (SOC under-diagnosis). Of the 75 SOC CDI cases that also had study testing, 12 (16%) were not study CDI cases (SOC over-diagnosis). SOC-undiagnosed and SOC-diagnosed CDI cases had similar demographics, medical histories, and clinical outcomes. Study testing identified 24% more CDI cases than SOC testing.

**Conclusion:**

There was a high incidence of hospitalized CDI in persons aged >50 years (154-202/100,000 PY). Of the hospitalized CDI cases, one-third were admitted to ICU and one-fifth died. Public health surveillance estimates of the incidence of laboratory-confirmed hospitalized CDI cases, which are based on SOC testing, may be under-estimated by 24%.

**Disclosures:**

**Frederick Angulo, DVM PhD**, Pfizer: Employee|Pfizer: Stocks/Bonds **Joann M. Zamparo, MPH**, Pfizer: Employee|Pfizer: Stocks/Bonds **Elisa Gonzalez, MPH**, Pfizer: Employee|Pfizer: Stocks/Bonds **Pingping Zhang, MS**, Pfizer: Employee|Pfizer: Stocks/Bonds **Michael W. Pride, PhD**, Pfizer: Employee|Pfizer: Stocks/Bonds **Sharon Gray, MPH**, Pfizer: Employee|Pfizer: Stocks/Bonds **Catia Matos Ferreira, PhD**, Pfizer: Employee|Pfizer: Stocks/Bonds **Forest W. Arnold, DO, MSc**, Gilead Sciences, Inc.: Grant/Research Support **Raul E. Isturiz, MD**, Pfizer: Employee|Pfizer: Stocks/Bonds **Nadia Minarovic, PhD**, Pfizer: Employee|Pfizer: Stocks/Bonds **Jennifer Moisi, PhD**, Pfizer: Employee|Pfizer: Stocks/Bonds **Luis Jodar, PhD**, Pfizer: Employee|Pfizer: Stocks/Bonds.

